# Hybrid STTR intervention for heterosexuals using anonymous HIV testing and confidential linkage to care: a single arm exploratory trial using respondent-driven sampling

**DOI:** 10.1186/s12889-015-2451-5

**Published:** 2015-11-16

**Authors:** Marya Gwadz, Charles M. Cleland, Noelle R. Leonard, Alexandra Kutnick, Amanda S. Ritchie, Angela Banfield, Holly Hagan, David C. Perlman, Talaya McCright-Gill, Dawa Sherpa, Belkis Y. Martinez, the BCAP Collaborative Research Team

**Affiliations:** Center for Drug Use and HIV Research (CDUHR), New York University College of Nursing, 433 First Avenue, 6th floor, New York, 10010 NY USA; Mount Sinai Beth Israel Medical Center, 120 East 16th Street, New York, NY USA

**Keywords:** Anonymous HIV testing, Heterosexuals, Health status disparities, Undiagnosed HIV, African American, Latino

## Abstract

**Background:**

An estimated 14 % of the 1.2 million individuals living with HIV in the U.S. are unaware of their status. Yet this modest proportion of individuals with undiagnosed HIV is linked to 44–66 % of all new infections. Thus innovative intervention approaches are needed to seek out and test those with undiagnosed HIV, and link them to HIV treatment with high retention, an approach referred to as “Seek, Test, Treat, and Retain” (STTR). The present protocol describes a creative “hybrid” STTR approach that uses anonymous HIV testing followed by confidential care linkage, focused on heterosexuals at high risk (HHR) for HIV, who do not test as frequently as, and are diagnosed later, than other risk groups.

**Methods/Design:**

This is a single-arm exploratory intervention efficacy trial. The study has two phases: one to seek out and test HHR, and another to link those found infected to HIV treatment in a timely fashion, with high retention. We will recruit African American/Black and Latino adult HHR who reside in urban locations with high poverty and HIV prevalence. Participants will be recruited with respondent-driven sampling, a peer recruitment method. The “Seek and Test” phase is comprised of a brief, convenient, single-session, anonymous HIV counseling and testing session. The “Treat and Retain” component will engage those newly diagnosed with HIV into a confidential research phase and use a set of procedures called care navigation to link them to HIV primary care. Participants will be followed for 6 months with objective assessment of outcomes (using medical records and biomarkers).

**Discussion:**

Undiagnosed HIV infection is a major public health problem. While anonymous HIV testing is an important part of the HIV testing portfolio, it does not typically include linkage to care. The present study has potential to produce an innovative, brief, cost-effective, and replicable STTR intervention, and thereby reduce racial/ethnic disparities in HIV/AIDS.

**Trial Registration:**

ClinicalTrials.gov, NCT02421159, Registered April 15, 2015.

## Background

More than three decades into the domestic HIV epidemic, over 55,000 individuals in the United States become infected with HIV each year, concentrated mainly among poor, stigmatized, and vulnerable populations [1]. African American/Black and Latino individuals are greatly over-represented in both incident and prevalent HIV cases [1] and substance use is a major risk factor for both HIV transmission and poor HIV health outcomes [2].

Of particular concern, an estimated 14 % of the 1.2 million individuals living with HIV in the United States are unaware of their HIV status [3]. Yet this modest proportion of individuals with undiagnosed HIV is linked to an estimated 44–66 % of all new HIV infections annually [4, 5], and uncovering this hidden group is an important public health priority. African Americans/Black and Latinos remain undiagnosed longer than Whites [2] and undiagnosed HIV is more common in males compared to females, and among heterosexual males compared to men who have sex with men (MSM) [2]. (Acronyms used in this protocol description are defined in Table 1.) In 2010, the National Institute on Drug Abuse (NIDA) at the National Institutes of Health called for research on new approaches to seek out persons with undiagnosed HIV, provide them with HIV counseling and testing, and then link those found to be HIV infected into medical care with high retention, which are referred to as “Seek, Test, Treat, and Retain” (STTR) studies (e.g., RFA-DA-11-001) [6]. The present protocol describes one such study focused on uncovering undiagnosed HIV infection among the population of heterosexuals at high risk (HHR) for HIV infection, who comprise 27 % of new HIV infections, but who are under-studied compared to other risk groups such as MSM and persons who inject drugs (PWID) [7]. The study focuses in particular on African American/Black and Latino HHR, who are concentrated in high- poverty urban geographical areas with a high local HIV prevalence, and who comprise the majority of HHR [8, 9]. Indeed, the Centers for Disease Control and Prevention (CDC) has called for research to test culturally appropriate interventions to overcome barriers to HIV testing and increase linkage to HIV care for heterosexuals in high-risk urban areas [10], where a generalized epidemic can be said to exist [11, 12].

**Table 1:** Acronyms used

ACASI	Audio Computer-Assisted Interviewing format
CDC	Centers for Disease Control and Prevention
HHR	Heterosexuals at high risk (for HIV)
HRA	High-risk area
ICER	Incremental cost-effectiveness ratios
MI	Motivational Interviewing
MRF	Medical Report Form
MSM	Men who have sex with men
NHBS	National HIV Behavioral Surveillance
NIDA	National Institute on Drug Abuse
OR	Odds Ratio
PWID	Persons who inject drugs
RDS	Respondent-driven sampling
RDS-ASTN	Anonymous single-session testing with navigation (using RDS)
RDS-CTTN	Confidential two-session testing with navigation (using RDS)
STTR	Seek, Test, Treat and Retain
VBS	Venue-based sampling

### Barriers to HIV testing and timely engagement in HIV care for HHR

African American/Black and Latino HHR experience barriers to regular HIV testing, and subsequent engagement in HIV care if found to be HIV-infected, at multiple levels of influence. The present study conceptualizes these barriers within the Theory of Triadic Influence [13]. The Theory of Triadic Influence is a social cognitive framework that emphasizes three “streams of influence” on health behavior: the individual/attitudinal, the social, and the structural. Among HHR, barriers to testing at the individual level of influence include lack of awareness of recommended testing frequency, and low perceived risk of HIV infection stemming from beliefs that HIV affects mainly PWID and MSM [14, 15]. Concurrently, fear of HIV testing and of a positive HIV test result are additional common individual-level barriers [16, 17], as is mistrust of medical environments [16]. Substance use is common among HHR and also serves as an impediment to HIV testing and linkage to care [16, 18]. Moreover, the population has other “competing priorities,” complicated by low socioeconomic status, such as mental health problems and unstable housing [14, 19]. At the social level of influence, the potential stigma of HIV testing and of a positive test result serve as barriers to HIV testing [20]. Moreover, peer norms regarding health care, including norms that regular HIV testing is not necessary for HHR, may impede testing [21]. At the structural level of influence, HHR have less access to settings where high-quality HIV testing is offered than their peers in the general underlying population [22, 23]. Theoretically, barriers at these three levels of influence interact to impede access to HIV testing and reduce motivation to test among HHR. Furthermore, this same set of barriers also impedes linkage to and retention in HIV primary care among HHR newly diagnosed with HIV [24–26]. The present protocol describes a culturally targeted STTR intervention strategy for HHR to reduce these barriers to HIV testing and timely engagement in HIV care.

### Our research team’s studies of STTR approaches

We recently published a protocol describing a study of two approaches for uncovering individuals with undiagnosed HIV infection [27]. One is a “Seek and Test” study using venue-based sampling (VBS) as its recruitment method. This approach provides confidential HIV counseling and testing in a single session in randomly selected public venues. The second approach is an STTR study using respondent-driven sampling (RDS) to enroll participants, followed by two intervention sessions (for engagement and training in how to recruit peers in the first session, and confidential HIV counseling and testing using a rapid HIV test provided in the second session), followed by care navigation to link those found HIV infected to health care in a timely fashion [27]. (The activities that make up the care navigation approach are described below.) RDS is a sampling methodology for studying subpopulations that are hard to define, reach, and/or engage. RDS is network-based method , similar to traditional snowball sampling, but with the goal of minimizing biases typically associated with those traditional methods [26, 28]. In RDS, a modest number of individuals are recruited directly by project staff (called “initial seeds”), and then trained to recruit a small number of their peers into the study. These peers then enter the study and peer recruitment continues until the sample size goals are met [26, 29]. The RDS method has four essential elements: tracking of recruitment chains; rationing of recruitment (usually 2–5 peers each); information on personal networks must be gathered (network size, recruitment refusals); and recruiters and recruits must have a pre-existing relationship [21]. We refer to this former study as VBS and the latter study as RDS-CTTN (“Confidential Two-session Testing with Navigation”). These two STTR studies are located in central Brooklyn, a location within New York City with high rates of poverty and highly prevalent HIV infection among heterosexuals, where African American/Black and Latino populations predominate [27]. In the present protocol we describe an innovative third STTR approach being evaluated in the same high-risk area in central Brooklyn, focused on the same vulnerable population of HHR. In contrast to the RDS-CTTN and VBS approaches which are providing confidential HIV testing, this “hybrid” approach begins with anonymous HIV testing in its “Seek and Test” phase, and then delivers intervention components to engage those found to be HIV infected in to a confidential “Treat and Retain” phase in order to link them to HIV care in a timely fashion, using care navigation.

### The pros and cons of anonymous testing

Anonymous HIV testing remains a critical part of the HIV testing portfolio. Anonymous testing is available in state and local health departments, in cases of occupational exposure, through home test/self-test technologies, and research studies including the National HIV Behavioral Surveillance (NHBS) system [30, 31]. There is some evidence to suggest individuals who test anonymously do so earlier in the course of their HIV disease than those who engage in confidential testing [32]. Additionally, anonymous testing may be useful for and/or preferred by individuals from marginalized populations [33]. As Kegeles and colleagues have found, many who seek anonymous HIV testing would avoid it under other circumstances, and anonymous testing may be preferred among those who suspect they are infected [34], and those who fear stigma and discrimination [35]. Moreover, in addition to being anonymous, the intervention described in the present protocol is designed to be brief and easy to access, with HIV counseling and testing conducted in a single efficient session. Thus, we hypothesize a brief, convenient, single-session, anonymous HIV testing effort may appeal to and engage HHR who face barriers to confidential and/or more labor intensive HIV testing.

Yet while anonymous testing may be an effective and/or efficient strategy in the ‘seek’ phase of STTR, it does not address the vital step of linkage to HIV care for those found to be HIV infected. In fact, although anonymous testing tends to yield individuals earlier in their HIV disease, those who test anonymously are more likely to experience delays entering care, because anonymous testing sites and programs do not typically have the resources to engage individuals into linkage programs and generally are not co-located with medical care [36, 37]. For example, in a study of characteristics of the HIV testing encounter and linkage to care, Reed [38] found that 36 % of those testing anonymously did not enter care within three months of diagnosis, compared with 26 % of those testing confidentially [38]. Similar to the RDS-CTTN study, participants in the present protocol are recruited using RDS, in order to access a largely vulnerable and hidden population of HHR, and we refer to the intervention described in this protocol as RDS-ASTN (“Anonymous Single-Session Testing with Navigation”).

### Study aims

This study has aims related to the Seek and Test as well as Treat and Retain project phases. In the Seek and Test phase, aims include estimating the proportion of HHR completing HIV testing, and the proportion found with previously diagnosed and newly diagnosed HIV infection. In the Treat and Retain Phase, aims include the feasibility of enrolling those newly diagnosed with HIV infection into a confidential research phase, as well as estimating the proportion successfully linked to care within three months. In both phases, participants in this protocol will be compared with participants recruited using the two other approaches to STTR (VBS and RDS-CTTN) described above and presented in detail elsewhere [27].

## Methods/Design

### Study setting in central Brooklyn

**Fig. 1:**
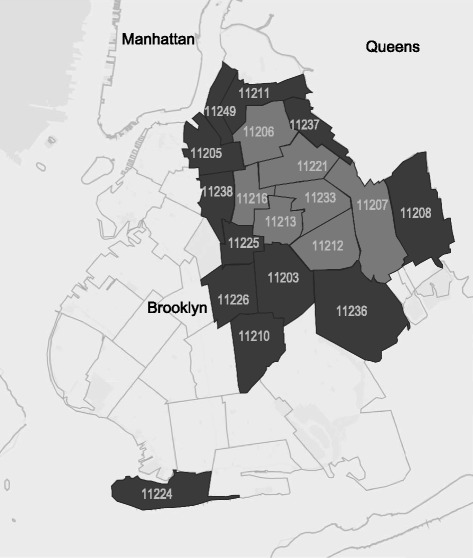
Core High-risk Area (HRA; in light grey) and surrounding larger HRA, in the borough of Brooklyn

Modeled on procedures developed for the NHBS system, the study will be conducted in a contiguous set of zip codes with the highest HIV prevalence and poverty rates in Brooklyn, NY, one of the five boroughs that make up New York City. We refer to this location as the “high-risk area” (HRA). The HRA is comprised of both a core HRA made up of seven contiguous zip codes with the highest rates of heterosexual HIV infection and poverty (the top 25 % of rank ordered zip codes), surrounded by an area made up of the second highest quartile of zip codes with respect to HIV prevalence and poverty (an additional 12 zip codes) (See Fig. 1). The procedures to select the HRA are described elsewhere [27]. In order to compare findings among the present study of RDS-ASTN and the VBS and RDS-CTTN studies, all three will be conducted in this same HRA. However, given the three studies’ aims to evaluate strategies to identify undiagnosed HIV infection, the present protocol is designed to avoid enrolling participants also enrolled in RDS-CTTN and thereby to tap into a different set of networks for RDS-ASTN. This is feasible because the HRA covers a large geographical area and the total population of the HRA is approximately 524,298 [39], mostly HHRs (Brooklyn’s total population is more than 2,600,000 [40]). Thus the present protocol includes procedures to reduce or eliminate cross-enrollment of participants who participated in RDS-CTTN into the present study, as we describe below. Those enrolled in VBS can participate in the present study, because VBS does not rely on networks for recruitment and VBS participants’ recruitment into RDS-ASTN via networks is consistent with the study’s larger goals, which include allowing network recruitment to proceed with relatively few restrictions.

### Design

**Fig. 2:**
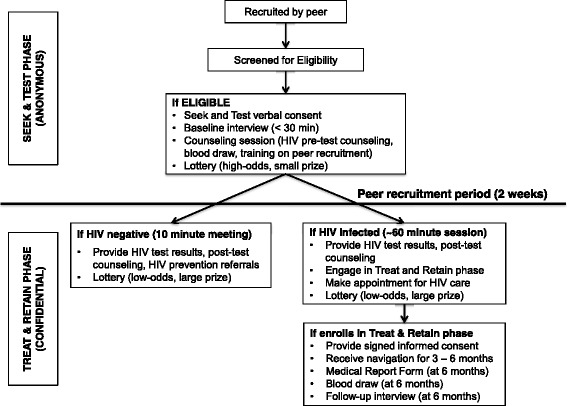
Primary activities conducted in the two-phase study

BCU is a single-arm exploratory intervention efficacy trial conducted in two parts. As noted above, participants are recruited using RDS. In the Seek and Test phase, participants recruited through peers present to the study, provide verbal informed consent, are screened, enrolled, and provided with anonymous HIV counseling and testing in a brief, single session, where a blood specimen is obtained for laboratory testing for HIV infection and results are provided 1-2 weeks later. In the Treat and Retain phase, a confidential research phase, those found to be HIV-infected in the Seek and Test phase are asked to give their names, provide signed informed consent, and engage in care navigation lasting 3–6 months to link them to HIV primary care in a timely fashion. Participants who present to the study with previous HIV diagnoses may enroll in the study, but do not receive HIV testing, as described below. However, they do have the opportunity to recruit peers. The study uses principles of behavioral economics and Motivational Interviewing (MI) to engage participants and increase their motivation to return for test results and enroll in the Treat and Retain phase if found HIV infected, described below. The protocol is registered with clinicaltrials.gov (NCT02421159) and the overall study design is presented in Fig. 2.

### Ethics statement

Study activities are approved by the Institutional Review Board at the New York University School of Medicine (OHRP #FWA00004952).

### Outcomes

The primary outcome of the Seek and Test phase is the relative yield (that is, proportion of newly identified HIV infections) of the RDS-ASTN intervention. We will assess the proportion of those newly diagnosed that engages in activities of the Treat and Retain phase (i.e., the feasibility of switching from an anonymous to a confidential phase). The primary endpoints for the Treat and Retain phase are the proportion linked to HIV primary care within three months (i.e., attending a care appointment and receiving CD4 and viral load tests) and time to the HIV care appointment. We will assess the acceptability and safety of the intervention components, as well as their cost-effectiveness. Because we examine many of these same endpoints in the other two components of the STTR study (VBS, RDS-CTTN), we can compare findings across the three STTR strategies.

### Study population and sampling considerations

An estimated 750 participants will be recruited by peers and screened, and 500 of these will be enrolled. An estimated 45 % of the sample will be female. An estimated 60 % of the sample will be African American/Black, 35 % Latino/Hispanic, and 5 % White/non-Hispanic. We estimate that > 50 % of the sample will be substance users. Given estimates of HIV incidence and prevalence in the local National Behavioral Surveillance System studies [41], we expect to identify 65 participants with HIV infection, 40 (8 %) newly diagnosed and 25 previously diagnosed with HIV.

### Hypotheses

The study will explore the following hypotheses: compared with VBS and RDS-CTTN, and controlling for potential differences on key socio-demographic characteristics across the samples, RDS-ASTN participants will have less HIV testing experience. Further, controlling for potential differences on key socio-demographic characteristics across the samples, RDS-ASTN will yield substantially higher rates of previously undiagnosed HIV compared with VBS and modestly higher rates compared to RDS-CTTN, although the RDS-ASTN intervention is less labor intensive than RDS-CTTN. This is because the brief and anonymous nature of the RDS-ASTN intervention may appeal to highly vulnerable individuals with multiple barriers to HIV testing. Further, we hypothesize that most participants (> 70 %) found to be HIV-infected in the RDS-ASTN intervention will engage in the Treat and Retain phase of the study, grounded in the efficacy of the MI approach combined with compensation based on behavioral economics principles to foster engagement. Last, we hypothesize that most participants in the Treat and Retain part (> 70 %), all of whom will be HIV infected, will be linked to care within 3 months, based on past studies of care navigation with similar populations [42, 43].

### Eligibility criteria

The study’s eligibility criteria are:

- 18–60 years of age
- sexually active (vaginal sex or anal sex) with at least one partner of the opposite sex within the previous year
- reside in the seven zip core central Brooklyn HRA (initial seeds only; peers may reside in the larger 19 zip code HRA)
- comprehend English or Spanish
- HIV negative or unknown HIV status (initial seeds only; peers may be ‘known positives’)
- willing to try to recruit peers
- not actively psychotic based on valid screening instrument
- not enrolled already in RDS-ASTN or in RDS-CTTN

Monolingual Spanish-speaking individuals are eligible for enrollment. All consent forms, recruitment, retention, assessment, and intervention materials will be translated into Spanish using the back-translation method [44]. Although the present study focuses on African American/Black and Latino/Hispanic populations, there is no race/ethnicity eligibility criterion to allow RDS recruitment chains to evolve with minimal restrictions. However, it is anticipated that > 95 % of participants will be African American/Black or Latino/Hispanic based on local population demographics and typical RDS recruitment patterns where peers tend to recruit racially/ethnically similar peers [45, 46].

### Individuals with previously diagnosed HIV infection

Participants who report they have previously been diagnosed as HIV infected during the screening interview, called “known positives,” may be enrolled in the study and may recruit peers. Indeed, HIV infected individuals may be an important link to those with undiagnosed HIV infection. HIV status will be confirmed with medical documentation by study staff by viewing one of the following: HIV diagnosis letter from a testing site; documentation of AIDS Drug Assistance Program coverage (a Federal program to provide HIV medications); a copy of the most recent HIV lab results or medical record including HIV diagnosis from a medical facility; current or recent antiretroviral therapy prescription or medication bottles; a signed and dated doctor’s note; or an M11Q, the New York State certification of an individual’s AIDS or HIV-symptomatic diagnosis. The type of documentation provided will be noted in a participant log. To preserve anonymity, the documentation will not be copied or retained by the study and no names or identifying information will be recorded. We have used these procedures to document HIV status in past studies [27, 46]. Study activities for those with known HIV infection are described below. In the next section we describe procedures for those who enter the study with an HIV-negative or unknown status.

## Seek and test phase (Anonymous)

### Study elements to foster participation, engagement, and retention

The study includes a number of components designed to build trust, engagement, and relationships, and to thereby encourage participation in study activities with high retention, while fostering a high quality experience for participants. First, the anonymous nature of the Seek and Test phase is designed to foster engagement. Second, study activities, including the intervention sessions, are grounded in the MI approach. MI is a flexible, collaborative counseling method that actively engages, focuses, and guides participants, without judgment or pressure, in order to elicit and strengthen durable, high quality intrinsic motivation for behavior change [47]. Consistent with this approach, participants’ choices and autonomy are respected, including if they decline to participate in study activities. Moreover, we will select study staff from diverse socioeconomic and racial/ethnic backgrounds, who enjoy working with substance-using and other vulnerable populations, and are knowledgeable about the concerns substance users and vulnerable populations experience, and who are expert in HIV testing and HIV care navigation. The study field site will be conveniently located in the HRA. Peer recruitment is another means of fostering engagement, because peers have credibility and peer recruitment is a type of endorsement of the project [26, 46, 48]. Last, as we describe below, compensation is structured using principals of behavioral economics to boost motivation to return for test results.

### Recruitment

Initial seeds who start recruitment chains (*N* = 2–20 initial seeds depending on recruitment rates) are recruited by study staff from outdoor and public venues in the neighborhoods located in the HRA; peers recruited by study participants will present to the study field site with a coded recruitment coupon that links the recruiter to the recruit, to track recruitment chains and compensate the recruiter.

## Consent

As described below, participants provide informed consent before engaging in study activities. In keeping with the anonymous nature of the “Seek and Test” study phase, verbal informed consent will be obtained. Participants entering the “Treat and Retain” study phase will provide signed informed consent.

### Screening for eligibility for the Seek and Test Phase

Verbal consent for a brief screening interview is obtained. Study staff will review the informed consent form for screening with potential participants and obtain their verbal informed consent for a brief, computerized screening interview to determine eligibility. Study staff will use a handheld or laptop computer to document the participant’s informed consent and then conduct the screening interview, which covers the eligibility criteria noted above. Individuals are given a copy of the screening informed consent form for their records. No names or identifying information will be collected from study participants; only unique identifiers will be used. Participants are compensated $15 for their time for this screening interview. Additionally, funds for local round-trip public transportation for all in-person encounters (assessments, sessions, navigation) are provided.

### Enrollment in the Seek and Test phase

Individuals who meet the eligibility criteria and are interested in participating in the Seek and Test phase will provide verbal informed consent to enroll into this phase, documented using a handheld/laptop computer. The consent form explains that this phase of the study is anonymous, and reviews study activities including a computerized baseline interview; an HIV pre-test counseling session; blood draw for HIV testing; use of blood samples for clinical lab tests (viral load, CD4), if found to be HIV infected; and a post-test counseling session to receive test results. Compensation levels are explained at this time. Individuals are given a copy of the informed consent form for the Seek & Test phase, and told that if they are found HIV-infected, the project will provide support to them as they adjust to their diagnosis. Participants are informed that, with their consent, if they are found to be HIV-positive, blood specimens will be tested for HIV viral load levels for research purposes without names, and that unused blood will be discarded. If their HIV test results are negative, all of their remaining blood specimens will be discarded. Individuals must agree to the baseline interview and counseling session to enroll in the study, which take place on the same day, but can decline the other components and continue in the study.

### Baseline interview

Participants then engage in a brief baseline interview (< 30 min) conducted in audio computer-assisted self-interviewing (ACASI) format. The assessment battery is comprised of a set of measures used in the National Institute on Drug Abuse (NIDA) funded STTR projects for vulnerable populations, assessing domains such as socio-demographic characteristics, HIV testing history, substance use frequency, substance use problems, and sexual partners [6, 49]. Participants are compensated $20 for their time for this assessment.

### Counseling intervention session and HIV testing

Individuals then complete a private one-on-one intervention/ counseling session led by a study staff member, lasting approximately 30–40 min, guided by the MI approach. The underlying goal of the first session is to engage participants into the study, build trust and relationships, and foster motivation to engage in study activities. The session consists of a series of brief exercises. First, participants are introduced to the study. The study rationale is described, and respect for participants’ choices and decisions throughout is emphasized. This includes the core message that if the participant tests positive for HIV, the study staff will help them get the high quality care they need, and if the participant tests HIV-negative, the study will provide them with referrals to help them stay that way. Second, the distinction between the anonymous Seek & Test study phase and the confidential Treat and Retain phase is briefly described, again with an emphasis on participants’ choices and autonomy. Third, HIV pre-test counseling is provided, consisting of a review of elements either required or recommended by the CDC and local Department of Health, such as a review of the nature of HIV and HIV transmission and risk reduction, review of the benefits of HIV testing and early treatment, explanation that HIV testing is voluntary, explanation of anonymous and confidential testing, and a review of confidentiality and discrimination protections and partner notification services. Next, participants receive a brief training on how to recruit peers for the study, and are encouraged to do so. This includes: who to recruit, how and when to approach peers, and how to refer peers to the project using a coded recruitment coupon. Then, a highly trained, experienced phlebotomist draws a blood specimen, which is sent to a laboratory for HIV testing, and a handout describing next steps in the study is reviewed, namely, the participant is asked to return to the project in approximately 7–14 days to collect compensation for peer recruitment (if any) and meet with the interventionist for post-test counseling and test results.

The blood specimen is transported using packaging mandated in the Federal Code of Regulations, CDC 42 CRF Part 72 and tested with the 4^th^ generation Alere assay (HIV-1/O/2 Antigen/Antibody Preliminary Test with Cascade Reflex to Supplementary Testing). The 4^th^ generation HIV test allows diagnosis of the earlier, acute phase (recent) of HIV infection, prior to the emergence of antibodies, effectively reducing the period after initial infection and before the detection of infection based on formation of detectable antibodies. The local Department of Health recommends the use of the 4^th^ generation HIV testing algorithm [50]. All work with human fluids is conducted at Biosafety Level 2. At this time, blood specimens are processed and stored for future HIV viral load testing if the participant tests positive for HIV. (It is not possible to store specimens for CD4 testing, so a second blood draw is conducted as appropriate in the second session.) Participants are compensated $15 for their time for this session, and $25 for the blood draw for HIV and viral load testing. They can complete the session and decline the blood draw and HIV testing.

### Building motivation to return for test results

The study includes a number of techniques to boost the proportion of participants who return for test results, which are necessary given that participants cannot be contacted or reminded about future activities. Drawing on work by Volpp and colleagues [51] that shows people are motivated by the experience of past rewards and the prospect of future rewards, participants in the first intervention session will enter a lottery with a good probability of winning a modest prize, namely, a 1 in 5 chance of winning $25, and a high probability of winning the smaller prize; that is, a 4 in 5 chance of winning $10 (a "high-odds, low-dollar" lottery) [51]. In the second session, participants will have a chance to participate in a "low-odds, high-dollar" lottery, with a 1 in 38 chance of winning a $50 prize, with the remainder receiving a low-dollar prize ($15). Second, participants are asked to address an appointment reminder card to themselves at the conclusion of the first intervention session, and place it themselves in a locked box, the contents of which are not visible to staff. Study staff will empty the cards from the lock box into a mailbox at the end of each day, without seeing any names or addresses. These retention methods will be explained in the consent form of the Seek and Test part. The study does not involve deception in any phase; all procedures are transparent.

### Peer recruitment period

After the intervention session, participants then enter the “peer recruitment period” of approximately 7–14 days during which they have the opportunity to recruit 2–6 peers to the study (depending on study recruitment patterns) using coded recruitment coupons that link the recruiter’s unique identification number to the recruit. When 3 or more coupons are distributed, at least one of the coupons is designated for females only, to boost the proportion of women in the study. Participants follow a recruitment script that instructs them to recruit individuals aged 18 – 60 years old; who they know by name or face, and who live in the larger HRA. Peers will be screened for eligibility, as described above. Recruiters receive $5 for each peer who presents for screening, plus a bonus of $10 for each eligible peer, for a total of $15 per eligible peer.

### Procedures to prevent duplicate enrollments

To prevent participants from enrolling in the present study (RDS-ASTN), and also in RDS-CTTN, the present has its own name (“Brooklyn Community United,” or BCU) and recruitment coupons have a different color than those used in RDS-CTTN. The screening interview assesses whether participants are enrolled in the RDS-CTTN study, and the participant’s unique identifier is checked against a master database. Participants are informed during the consent process they may be withdrawn from the study if they have been previously enrolled in RDS-CTTN. Initial seeds to start peer recruitment will be recruited from the same HRA as the RDS-CTTN participants, but from the zip code with the fewest number of RDS-CTTN participants, as a means of tapping into network recruitment chains of individuals with no exposure to RDS-CTTN. Although we may not be able to eliminate duplicate enrollments, these procedures will greatly reduce their occurrence.

## Treat and Retain Phase (Confidential)

### Post-test counseling session for all participants

Participants return for their HIV test results approximately two weeks after the first session, and if found to be HIV infected, are presented with the opportunity to enroll in the Treat and Retain phase to receive support as they adapt to the new diagnosis, and link to HIV care in a timely fashion. They also receive compensation for peer recruitment at this time, which may encourage attendance at this appointment. (They can receive compensation for peer recruitment and decline to participate in the second session, however.) The session begins with elements either required or recommended by the CDC and local Department of Health for post-test HIV counseling. For participants whose test results are negative, the interventionist will address personal HIV risk/harm reduction; note CDC recommendations for annual testing; and will provide verbal instructions or a written referral directory for community-based HIV prevention services and other services (mental health care, substance use, housing). This session is brief (< 10 min) and will be the last study activity for HIV-negative study participants. Participants are compensated $15 for their time for this session.

For those who are found newly infected with HIV, the session is designed to provide post-test counseling. In addition, it will foster engagement into the Treat and Retain phase through demonstrated expertise of the interventionist with respect to HIV and HIV primary care, and continued use of the MI approach to build motivation to accept care navigation and support. This session will last approximately 60 min. Regarding post-test counseling, the interventionist will: provide the positive test result, and elicit and discuss reactions to the diagnosis, with a discussion of typical next steps for newly diagnosed individuals including adjustment, limited disclosure, HIV primary care, and reducing sexual and injection-related risk behavior. The interventionist will provide psychological support, ensure that the client has accurate information about steps necessary to prevent transmission; discuss with the participant whom to notify of his/her positive test result; offer assistance with short-term planning to cope with new diagnosis and emphasize the importance of HIV care. As a central aim of this study is to facilitate transition to the confidential Treat and Retain phase, which involves relinquishing anonymity, a portion of the session will be devoted to explaining this phase. To foster engagement, culturally targeted core messages will include: framing the problem of delayed linkage to care as a community issue (highlighting both racial/ethnic disparities in linkage to care and HIV outcomes, and progress made in linkage among populations of color); discussing multi-level barriers that individuals experience to HIV care, related to gender, substance use, low socio-economic status, and/or race/ethnicity; explaining what “confidential” means, including laws regarding reporting of names of newly diagnosed individuals to the local Department of Health (which is mandatory in our jurisdiction); providing handouts with activities and compensation; reviewing a menu of options and evoking benefits of each; and determining if the participant wishes to enroll in the Treat and Retain phase. All participants have the opportunity to engage in the lottery activity at this point.

Finally, we will make an HIV care appointment for the participant regardless of whether or not he/she enrolls in the Treat and Retain phase, consistent with local public health law. Participants who have not given their full name to the study can be put on the phone directly with the HIV care site and provide that information directly to the facility.

### Enrollment into the Treat & Retain Phase

Participants who are interested in enrolling in the Treat & Retain phase will next enter that phase of the study, beginning with signed informed consent and completing a locator form. Participants who are unsure whether they wish to enroll have a “grace period” of approximately three weeks to decide. They can contact the study to schedule an enrollment visit within that period. Compensation of $15 is provided for the second session.

### Care navigation

 Care navigation is a flexible and individualized intervention approach to reduce disparities in care for low income or marginalized populations [52, 53]. In practice, care navigation identifies and resolves the organizational, social, and individual barriers patients may experience to accessing services [54]. We will provide care navigation to newly diagnosed HIV-infected participants over a three- to six-month period, depending on the participant’s needs. During navigation, participants will be aided to identify and overcome barriers that they may encounter at various steps during the course of their linkage to HIV care. Navigation will consist of face-to-face meetings and phone contacts to provide participants with support, assistance making decisions about disclosing their HIV status, referrals, coordination of care, accompaniment to medical appointments, and assistance to get other necessary services or care. Only a limited number of face-to-face meetings will be compensated ($10, up to 5 compensated meetings in the first month) so as to not artificially increase navigation “dose.” Participants will be encouraged to contact the study outside of scheduled care navigation meetings/contacts for additional help or support, as needed. Duration and content of care navigation contacts are based on individual participant needs and will vary across participants, and will be logged to document their duration and content. We estimate that > 70 % of participants will be engaged in care by the end of three months. Those who are not engaged have the option of extending the care navigation period for an additional 3 months, for a total of 6 months of care navigation.

### Additional recruitment coupons

Individuals who test HIV-positive have the opportunity to recruit three additional peers during the care navigation phase, a type of network-based case finding [55, 56]. They will be encouraged to recruit their sexual and injection drug using partners, if they have not done so already.

### Medical Report Form and blood draw

At the end of the 6-month Treat and Retain phase, we will ask participants to have a Medical Report Form (MRF) completed by their HIV care provider. The Medical Report Form will contain health care appointment data abstracted from their medical chart, and will be signed by a health professional at the facility where the participant receives care. Participants can request that the form be filled out and return it to us, for which they will receive compensation ($25). Alternately, participants can request for us to contact the provider directly to obtain the data either over the phone, by secure fax or in person. Further, study staff will obtain a second set of blood specimens for CD4 and HIV viral load testing at the end of 6 months, and participants will be compensated $25 for this blood draw.

### Follow-up interview

At the end of the 6-month care navigation period, participants will be asked to participate in a brief follow-up interview assessing the health, relationship, and substance use indices assessed in the baseline interview, but focused on the past 6 months, as well as safety (social harms) and intervention acceptability. The follow-up interview will be conducted using the ACASI format ($30 compensation).

### Procedures for participants who are “known positives” (not shown on Fig. 2)

Individuals who report being HIV-infected during the screening interview and who meet the study’s eligibility criteria will provide verbal informed consent for and be enrolled in a track of the Seek and Test phase of the study. These participants will be asked to provide medical documentation of their HIV-positive status (which will be viewed but not retained); they will engage in a baseline interview that includes questions on their HIV history (e.g., date of first diagnosis with HIV), and receive a brief intervention session to orient them to the study and train them how to recruit peers. They will not be tested for HIV or receive other lab tests. Individuals who cannot provide medical documentation of their HIV-status will be enrolled in the main Seek and Test phase (described above), and offered participation in all of those activities. These HIV-infected participants who are not well engaged in care by DHHS definition [57] will be given the opportunity to enroll in the Treat and Retain phase of the study, for which they will provide signed informed consent. They will be asked to complete a Medical Report Form at the beginning and end of the Treat & Retain phase and will be provided with care navigation over three months. Compensation will be provided at the rates described above.

### Analysis plan

First, we will compare the yield of RDS-ASTN with the two other STTR strategies we described in a separate protocol, RDS-CTTN and VBS [27], with respect to newly diagnosed HIV infection. We will examine differences in demographic characteristics between VBS, RDS-CTTN and RDS-ASTN participants and adjust for these when comparing STTR approaches. Logistic regression will be used to compare the three STTR strategies on yield of newly diagnosed HIV infection. Covariates will include prior HIV testing.

Second, we will explore the feasibility of engaging RDS-ASTN participants who test HIV-positive into a confidential Treat and Retain study phase. Key indicators of feasibility will include the percentage of participants with HIV infection who accept the transition from the anonymous testing phase to the confidential Treat and Retain phase; and rates of engagement in Treat and Retain phase study activities. We also will explore the efficacy of the Treat and Retain components in terms of time to an HIV clinic appointment among newly identified HIV-infected HHR. We will describe the occurrence and timing of a first HIV clinic appointment and whether viral load and CD4 results were obtained. Cox proportional hazards regression will be used to compare STTR strategies (RDS-CTTN and RDS-ASTN) on the occurrence and timing of first HIV clinic appointment.

Third, we will project the clinical impact, costs, and cost-effectiveness of RDS-ASTN for identifying HHR, and linking them to and retaining them in HIV-related medical care. Incremental cost-effectiveness ratios (ICER) will be presented in terms of costs per HIV infection prevented and used to compare STTR strategies. We will estimate the costs and effectiveness outcomes for ICERs with projections from a mathematical model of HIV infections prevented, based on reductions in HIV risk behavior [58, 59] and viral load suppression associated with treatment [60, 61]. For outcomes not directly observed in participants recruited by RDS-ASTN, we will assume these are the same as for the RDS-CTTN intervention group; that is, an RDS-ASTN participant linked to care and retained for three months has the same probabilities of ART initiation and viral load suppression as an RDS-CTTN participant. The time horizon for our analysis is lifetime.

Finally, generalized linear models will be used to compare the socio-demographic, substance use, health, and HIV testing characteristics of those enrolled by RDS-ASTN with those in RDS-CTTN and VBS. This analysis will determine whether there are important differences in risk behavior and HIV testing history among these study groups, which may indicate differential effectiveness in reaching higher risk heterosexuals and those who have not tested or have tested infrequently.

### Power analysis

The study is powered to detect a difference in Seek and Test approaches between RDS-ASTN and VBS of odds ratio (OR) OR = 2.92 and between RDS-ASTN and RDS-CTTN of OR = 1.71, both with 80 % power (α = .05). Power will be approximately 80 % to detect a small effect size (*d* = .19) when comparing socio-demographic, substance use, health, and HIV testing characteristics across STTR approaches.

### Limitations

The proposed study lacks a control condition that is similar to the planned RDS-ASTN strategy but which does not offer care navigation for those with HIV infection, as all HIV-infected participants will receive care navigation. We considered randomly assigning seed participants and their recruits to RDS-ASTN with and without navigation to care, but because we are primarily concerned with comparing the yield of newly diagnosed infections across STTR strategies, we chose to include the care navigation intervention for all ASTN participants. Moreover, a condition with brief, single-session anonymous testing and without care navigation may not meet the standard of equipoise. Another limitation is the brevity of follow-up; however, it will allow us to obtain what we consider to be the two most important outcomes needed to determine the feasibility and potential of this STTR strategy: engagement in the confidential Treat and Retain phase, and linkage to a first HIV clinic appointment. Last, the two Seek and Test approaches in the main study and the novel approach to be examined in the present study all compensate participants for study activities. Although compensation is similar across groups, the yield in all groups may be higher than what could be achieved without compensation for these activities.

### Generalizability

In this study, reproducibility is more important than generalizability. Although there are imperfect methods for weighting RDS data to obtain representative samples, this study does not seek to draw inferences based on representativeness. Rather, the methods we propose to recruit a population of HHR with excess rates of undiagnosed HIV should be reproducible, that is, application of the same methodology in other settings should also recruit a high-risk sample. This has been demonstrated to some degree in NHBS. When the sample was restricted to individuals living in urban HRAs where, as is characteristic of all New York City HRAs, > 20 % of residents lived below the poverty line, and HIV prevalence was 2.1 % - a rate that also meets the definition of a generalized HIV epidemic [10]. Thus, evidence exists to support the likelihood that the recruitment and intervention method shown to yield the largest proportion of HIV-positive HHR, and for which subsequent timely linkage to care is reasonably successful, could produce similar results in other impoverished urban settings.

## Discussion

The present study seeks to examine an innovative anonymous method to uncover undiagnosed HIV infection in an understudied population: heterosexuals at high risk for HIV. Further, it tests components to link those with HIV infection to HIV primary care with high retention, both newly diagnosed and those with previous HIV diagnoses. Results can guide the addition of anonymous testing with effective care linkage to the currently available portfolio of STTR strategies, broadening the scope of testing and care linkage to unknown HIV infected individuals for whom confidential testing is not acceptable, and who may otherwise never be linked to care. Study results will provide guidance on the most efficient and cost-effective means of uncovering this largely hidden and vulnerable population. The ultimate aim of the present study is to provide an efficient, cost effective, reproducible, and scalable sampling method and intervention approach to address the critical public health problem of undiagnosed HIV infection and delays in engagement in HIV care among HHR. This protocol provides background for other investigators interested in researching this population, which is challenging to define, reach, and engage. We hope to be able to delineate characteristics (demographic, attitudinal, experiential) of those who are averse to confidential testing, but willing to engage in anonymous testing with subsequent care linkage; these findings can inform targeted anonymous testing efforts which could potentially have a significant impact on the HIV burden in affected communities.
